# Homology-Based Variant-Effect Predictors Break Down on Cytochrome P450 Pharmacogenes*

**DOI:** 10.64898/2026.07.30.741616

**Published:** 2026-08-05

**Authors:** Helen Xu, Issah Samori, Gowri Nayar, Russ B. Altman

**Affiliations:** 1 School of Medicine, Stanford University, Stanford, CA, USA; 2 Department of Bioengineering, Stanford University, Stanford, CA, USA; 3 Department of Biomedical Data Science, Stanford University, Stanford, CA, USA; 4 Department of Genetics, Stanford University, Stanford, CA, USA

**Keywords:** Cytochrome P450, variant-effect prediction, pharmacogenomics, protein language models, deep mutational scanning

## Abstract

Cytochrome P450 (CYP) enzymes metabolize roughly three-quarters of clinically used drugs; genetic variation in these enzymes is a leading source of interindividual differences in drug response. Predicting a variant’s functional effect is therefore critical, yet the consequences of most CYP variants remain unknown. Many state-of-the-art variant-effect predictors rest on a homology-based paradigm that scores variants by evolutionary conservation — an assumption that pharmacogenes including CYPs violate. Indeed, focusing on human CYPs, we show that homology-based models fail systematically. AlphaMissense (AM) assigns variants to its “ambiguous” class at nearly twice the proteome-wide rate across six CYPs, and within that class the scores are essentially uncorrelated with CYP2C9 DMS activity (Spearman’s *ρ* = 0.069); Evolutionary Scale Modeling 2 (ESM-2) shows the same pattern. We further hypothesized that non-homology-based features (sequence position, substitution chemistry, binding-site distance, and secondary structure) might help resolve the ambiguous calls, but their explanatory power is weak. Using CYP2C9 DMS activity as ground truth, we built a *k*-nearest-neighbors model over ESM-2 embeddings and ensembled it with AM and ESM-2 masked marginal probability, improving the ambiguous-class correlation roughly ten-fold, from *ρ* = 0.069 to 0.715 (overall *ρ* from 0.638 to 0.825). However, this markedly improved accuracy does not translate into agreement with clinical annotations. Drawing on evidence that a variant’s effect can depend on the drug, we hypothesize that substrate identity is the key missing feature in current models, and that predicting function for these multi-substrate enzymes may require redefining function as substrate-conditioned.

## 1. Introduction

Adverse drug reactions are among the leading causes of death in hospitalized patients. Much of this harm is not idiosyncratic but genetic: interindividual differences in the enzymes that metabolize drugs mean the same dose can be therapeutic for one patient and fatal for another.^1,2^ Chief among these enzymes are the cytochrome P450s (CYPs), which are involved in the metabolism of roughly three-quarters of all clinically used drugs.^3–5^ Genetic variation in CYP genes is correspondingly one of the largest sources of interindividual variability in drug response.^6,7^ CYP2C9 alone metabolizes up to ~15% of small-molecule drugs, including the widely prescribed anticoagulant warfarin, for which variant-driven differences in metabolism directly affect safety and efficacy.^8^ Pharmacogenomic testing and genotype-guided prescribing aim to pre-empt these outcomes,^9,10^ but in practice they rest on a small set of well-characterized “star” alleles. The functional consequences of the vast majority of observed CYP variants remain unknown, so we generally cannot predict whether a given variant will increase, decrease, or leave unchanged the metabolism of a specific drug. Closing this prediction gap is a prerequisite for scaling pharmacogenomics beyond its current handful of actionable alleles.

The number of possible variants vastly exceeds what deep-mutational, biochemical, or clinical characterization can hope to interrogate, so filling this gap necessarily requires computational variant-effect prediction. Yet most widely used predictors annotate a variant by its similarity to sequences shaped by evolution — the same homology-based paradigm that computational function annotation has relied on for decades. AlphaMissense (AM) adapts AlphaFold and is fine-tuned on human and primate population-frequency data to predict pathogenicity at high precision against clinical ClinVar benchmarks.^11,12^ Protein language models of the Evolutionary Scale Modeling (ESM) family are trained by masked-language modeling over millions of divergent sequences. General-purpose members such as ESM-1b^13^ and ESM-2^14^ are not inherently variant-effect predictors (VEPs) but can be repurposed as zero-shot predictors through their masked marginal probabilities (MMP);^15^ ESM-1v,^16^ by contrast, was purpose-built for zero-shot variant-effect prediction. Related evolutionary and generative models likewise infer mutational effects from sequence patterns alone.^17^ Whether the alignment is explicit, as in AM, or learned implicitly across a pretraining corpus, as in ESM-2, both approaches ultimately score a variant based on the residue preferences that natural selection has imposed on a position across homologs.

This assumption works well for the disease genes these models are usually tuned on: such genes are under strong evolutionary pressure to stay unchanged, so a variant that breaks conservation is likely to be harmful. Yet pharmacogenes violate these assumptions. CYPs evolved largely to metabolize compounds in the diet and environment, and their central role in drug metabolism is in a sense incidental to that history.^18,19^ As a result, reduced or even absent activity of an individual CYP is frequently benign on its own, with consequences that surface only when a particular food or drug is encountered. CYP variants are therefore usually not disease-causing but instead modulate drug metabolism; they frequently occur at high population frequencies; and they arise in a gene family that is unusually polymorphic and functionally diverse rather than strongly conserved.^4,6^ Consistent with this mismatch, homology- and pathogenicity-oriented predictors perform markedly worse on pharmacogenetic than on disease variants, motivating a growing body of pharmacogene-specific methods and evaluations.^20–24^ What has remained unclear is whether this degradation reflects mere low model confidence or a genuine information ceiling, in which an important determinant of function is missing entirely and cannot be recovered by better calibration alone.

Among pharmacogenes, CYPs are one of the more tractable settings for examining this, combining these violated assumptions with clinical necessity and a rich, though still incomplete, body of ground truth. Decades of biochemical, structural, and pharmacogenetic work have produced variant-level data spanning high-throughput deep mutational scanning (DMS) of enzymatic activity and abundance,^8,25^ clinical annotations, and curated pharmacogenomic databases, yet the functional consequences of most CYP variants are still unresolved. Because CYPs are comparatively well-characterized, cases where conservation-based predictors fail more plausibly expose a limit of the homology paradigm than an absence of data.

In this work, we use human CYP pharmacogenes as a case study for where and why homology-based variant-effect prediction breaks down. Our contributions are:

1. We quantify a systematic failure of homology-based VEPs, reproducible across six CYPs and localized to the “ambiguous” class of AM predictions, where scores are essentially uncorrelated with measured enzymatic activity.
2. We show that biologically grounded features (sequence position, substitution chemistry, distance to the binding site, and secondary structure) only weakly account for model failure.
3. We show that a k-nearest-neighbors model over ESM-2 embeddings, in an ensemble with AM and ESM-2 MMP, recovers most of the lost signal against experimental data for CYP2C9 (where DMS activity data are available), yet this accuracy does not translate into reliable agreement with clinical or pharmacogenetic labels.
4. We propose substrate identity as the leading candidate for the missing, non-homologous variable, drawing on pharmacogenetic evidence that a single variant’s consequence can depend on the drug, and argue that substrate-conditioned prediction is a promising route beyond homology.

Taken together, these results reframe the annotation problem for pharmacogenes: the barrier is not simply that homology-based similarity is too weak, but that the definition of function these models predict, a single substrate-agnostic effect, is the wrong target for multi-substrate enzymes. Moving beyond homology here may therefore require redefining function itself, as a quantity conditioned on substrate, rather than more or better similarity search.

## 2. Methods

### 2.1. Datasets and variants

We drew on four ground-truth resources, each encoding a distinct notion of variant effect. DMS activity measurements were taken from Amorosi et al. (2021),^8^ which reports click-seq (enzymatic activity) scores for N=6,527 CYP2C9 missense variants. We used the normalized click-seq activity score as the primary quantitative target throughout. ClinVar provided disease-oriented clinical significance labels following the ACMG framework;^26,27^ after filtering for CYP2C9 single-missense variants with a functional classification, 50 variants at 48 unique positions remained. PharmVar provided pharmacogenetic function classes;^28^ we retained the 77 CYP2C9 star alleles defined by a single missense substitution with functional classification. ClinPGx (PharmGKB) provided literature-curated variant–drug association annotations used for the substrate-resolved analysis (Section 2.3.5).^29^

The enrichment analysis (Section 2.3.1) and substrate-resolved analysis (Section 2.3.5) spanned six clinically significant CYPs (CYP1A2, CYP2C19, CYP2C9, CYP2D6, CYP2E1, and CYP3A4) whereas activity-benchmarked modeling focused on CYP2C9, for which comprehensive DMS activity data were available. Reference protein sequences for all CYPs were obtained from UniProt.^30^ All analysis was done in Python 3.12.11 on the Sherlock computing cluster operated by Stanford Research Computing.

### 2.2. Variant-effect predictors and models

#### 2.2.1. Predictors and model selection

We evaluated three predictors. For CYP2C9 (UniProt P11712) and the five additional CYPs, we obtained AM pathogenicity scores along with AM’s discrete class labels (likely benign, ambiguous, or likely pathogenic), which AM assigns by thresholding its predicted pathogenicity score. ESM-2 was used as a zero-shot variant-effect signal via its MMP (the difference in masked log-likelihood between mutant and wild-type residue, $\log\;P(x_{i}=\text{mut}\;|\;x_{\backslash i})-\log\;P(x_{i}=\text{wt}\;|\;x_{\backslash i}))$, and as protein embeddings for a *k*-nearest-neighbors model (Section 2.2.3). We selected ESM-2 over ESM-1v for the main analyses because ESM-2’s zero-shot scores tracked measured CYP2C9 activity more closely (Fig. S1).

#### 2.2.2. Score-to-activity calibration

Because AM and ESM-2 MMP scores are not on the activity scale, we calibrated each to click-seq activity using monotone regression. For each signal we compared isotonic and sigmoid calibrators by 5-fold out-of-fold (OOF) *R*^2^ and selected the better-performing option: isotonic regression for AM (monotone decreasing as higher pathogenicity corresponds to lower activity), and four-parameter monotone-increasing logistic (sigmoid) fit for ESM-2 MMP.

#### 2.2.3. CYP-specific KNN over ESM-2 embeddings

We built a *k*-nearest-neighbors (KNN) regressor predicting click-seq activity from ESM-2 embeddings. Each mutated CYP2C9 protein, also referred to as a variant, was represented by the final-layer, per-residue ESM-2 representation, pooled over all sequence positions (excluding the BOS/EOS tokens) to a 1,280-dimensional vector. Hyperparameters — residue pooling, neighbor weighting, distance metric, and the number of neighbors K — were tuned against activity using Spearman correlation between OOF predictions and measurements under 5-fold cross-validation (Fig. S2). The optimal configuration (mean pooling, cosine distance, distance-weighted averaging, K=5) was used in all subsequent analyses.

#### 2.2.4. Ensemble model

We combined the calibrated AM activity, calibrated ESM-2 MMP activity, and KNN-predicted activity using a ridge-regression meta-learner on standardized features (StandardScaler followed by RidgeCV over *α* ∈ [10^−3^, 10^3^]). To prevent leakage, the base-learner predictions used as meta-features were generated by inner 4-fold cross-validation within each training fold, and all models were evaluated by outer 5-fold cross-validation, reporting OOF Spearman’s *ρ* against click-seq activity. A simplex-constrained weighted average gave near-identical performance, so we report the ridge ensemble throughout.

### 2.3. Statistical analyses

#### 2.3.1. Ambiguous-class enrichment across CYPs

For each of the six CYPs we retrieved all possible missense variants and their AM classes, and compared the fraction assigned to the ambiguous class against the proteome-wide baseline reported by the original AM paper.^11^ Significance was assessed with a one-sided binomial test against the proteome-wide ambiguous rate.

#### 2.3.2. Within-class score–activity correlation

A predictor can achieve a strong overall correlation with activity simply by separating variants into low- and high-activity groups, even if it cannot accurately rank variants within any group. To distinguish these cases, we computed Spearman’s *ρ* between each predictor score and click-seq activity within each AM class (likely benign, ambiguous, likely pathogenic). Differences between the ambiguous-class correlation and the other classes were tested with a Fisher r-to-z test for independent correlations.

#### 2.3.3. Structural and biochemical priors

We tested four interpretable features for association with AM class. **(1)** *Positional distribution* asks whether the AM classes cluster in particular regions of the protein sequence: per-position enrichment of each class, tested by per-bin *χ*^2^ goodness-of-fit with Benjamini–Hochberg (BH) correction. **(2)** *Substitution chemistry* asks whether an AM class is associated with the biochemical nature of the amino-acid change: enrichment across (wild-type, mutant) amino-acid pairs, with residues grouped as nonpolar (GAVLIMPFW), polar (STCYNQ), positively charged (RKH), or negatively charged (DE); a *χ*^2^ test assessed association of AM class with conservative (within-group) versus radical (cross-group) substitutions, and we hierarchically clustered (Ward linkage) wild-type and mutated residues by their per-substitution ambiguous-fraction profiles. **(3)** *Binding-site proximity* asks whether an AM class is associated with distance from the catalytic center: the *Cα* distance from each variant to the annotated binding-site residue 435 (the axial cysteine) on the AlphaFold structure,^12^ binned and tested by *χ*^2^ goodness-of-fit with BH correction. **(4)** *Secondary structure* asks whether the AM classes are enriched in particular structural elements of the protein: elements assigned with SecStrAnnotator^31^ and tested for class enrichment by Pearson’s *χ*^2^ test of independence.

#### 2.3.4. Clinical and pharmacogenetic benchmarking

We compared model-predicted activity against the held-out ClinVar and PharmVar labels (Section 2.1), which were excluded from ensemble training, using Spearman’s *ρ* between predicted activity and the ordinal functional labels, where higher values denote greater retained function. PharmVar comparisons were additionally stratified by evidence level (definitive; definitive + moderate; all known function alleles). Note the limited available data: only seven CYP2C9 missense variants both carry a non-uncertain ClinVar label and are present in the DMS activity data, and PharmVar evidence tiers range from n=9 (definitive) to n=27 (all known function) alleles.

#### 2.3.5. Substrate-resolved analysis

For the substrate-resolved analysis, ClinPGx variant–drug annotations for all six CYPs were deduplicated to the best-evidence per (variant, drug) pair and restricted to variants tested against at least two of each gene’s twelve most-frequent drugs. We tested substrate dependence at two levels: a per-variant *χ*^2^ test of function label against drug (BH-corrected across variants within each gene), and a gene-level Cochran–Mantel–Haenszel test (function labels collapsed to a binary reduced-versus-not outcome) asking whether a given drug affects function while holding variant identity fixed.

## 3. Results

### 3.1. AM fails systematically on CYPs, not randomly

**Fig. 1.**
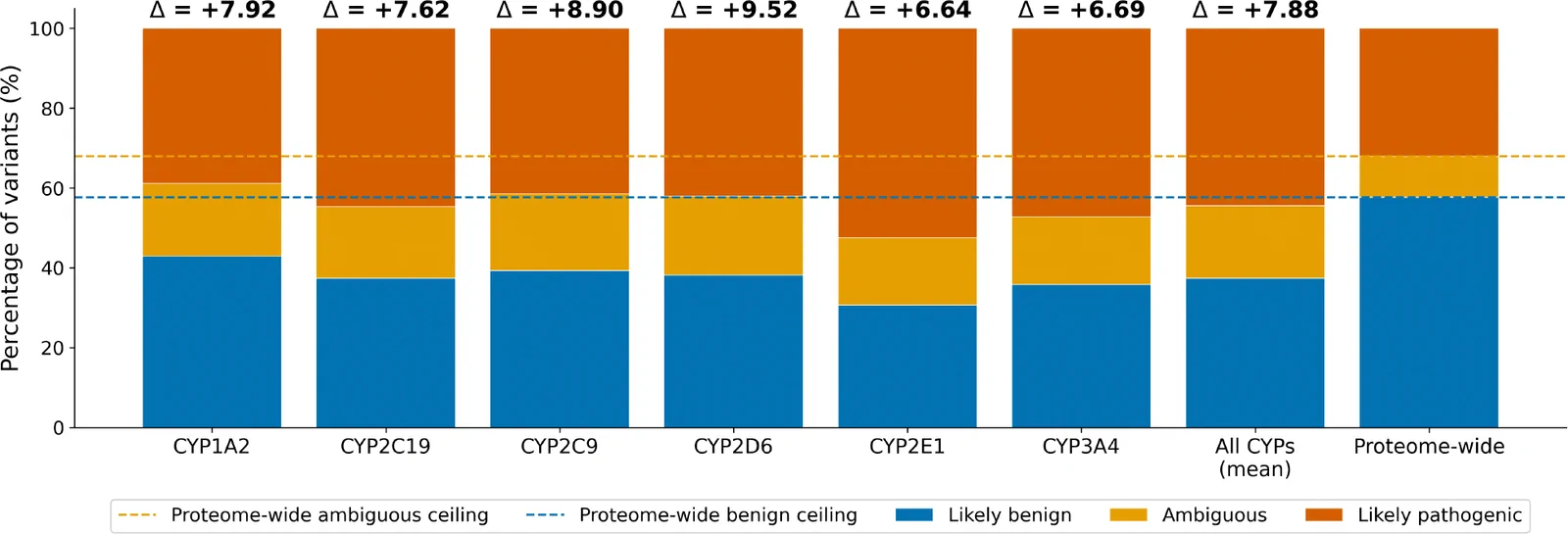
Rate of AM ambiguous pathogenicity labels in CYPs versus the proteome-wide baseline.^11^ Each Δ above a bar is the difference (in percentage points) between that bar’s ambiguous fraction and the proteome-wide ambiguous fraction. Dashed lines mark the proteome-wide ambiguous and likely benign ceilings.

AM assigns CYP missense variants to its ambiguous pathogenicity class far more often than the proteome as a whole. Pooling all possible missense variants across the six CYPs (N=56,677 variants), 18.161% were classified as ambiguous, versus a proteome-wide baseline of 10.282% reported by AM^11^ — a 1.766-fold enrichment that is highly significant by a one-sided binomial test (*p* < 10^−16^) and consistent across all six CYPs examined (Fig. 1). This is therefore not an artifact of one CYP but a family-wide property.

### 3.2. AM’s signal is class-dependent: strong across classes but weak within them, and absent in the ambiguous class

**Fig. 2.**
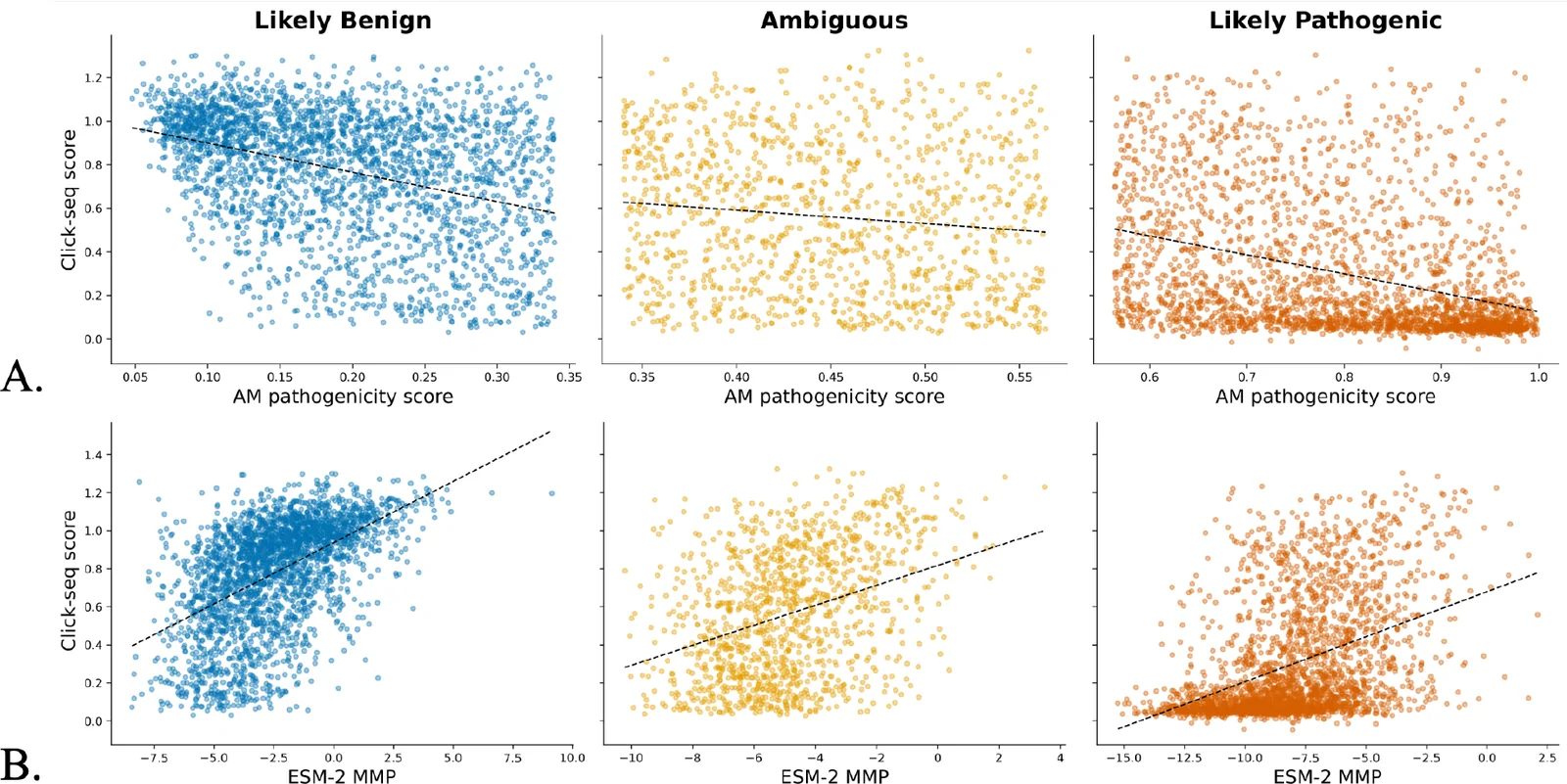
Predicted AM pathogenicity score (**A**) and ESM-2 MMP (**B**) versus CYP2C9 DMS click-seq activity, stratified by AM class (likely benign, ambiguous, and likely pathogenic). Each point is one missense variant, and dashed lines indicate ordinary least-squares linear fits.

To determine whether AM remains informative for CYPs despite the elevated ambiguous rate, we correlated its pathogenicity score with measured click-seq activity within each of the classes. The correlation is moderate within the likely benign (Spearman’s *ρ* = 0.339) and likely pathogenic (*ρ* = 0.414) classes but collapses within the ambiguous class (*ρ* = 0.069, i.e. essentially no activity signal) (Fig. 2A). A Fisher r-to-z test confirms that the ambiguous-class correlation is significantly weaker than both the benign (*p* = 5.66×10^−12^) and pathogenic (*p* < 10^−16^) classes. AM’s respectable overall pooled correlation (*ρ* = 0.638; Table 1) is thus manufactured largely by separation between the classes rather than by ranking variants within them, and is uninformative within the class it classifies as ambiguous.

ESM-2, used as a zero-shot predictor through its MMP, exhibits the same pattern, though less severely: its within-class Spearman correlations are 0.544 (benign), 0.340 (ambiguous), and 0.445 (pathogenic), with the ambiguous class again significantly weaker than benign (*p* = 2.98 × 10^−13^, Fisher r-to-z test) and pathogenic classes (*p* = 2.72 × 10^−4^) (Fig. 2B). That two widely used predictors built on similar conservation-based signals fail in the same place indicates a shared failure of the homology paradigm rather than an idiosyncrasy of one model.

### 3.3. Biological priors only weakly explain which variants are classified as ambiguous

If the ambiguous class were structurally or biochemically coherent, some interpretable features should distinguish it from the benign and pathogenic classes. Across four such priors, each carries some class structure, but none cleanly distinguishes ambiguous from benign and pathogenic variants.

**Fig. 3.**
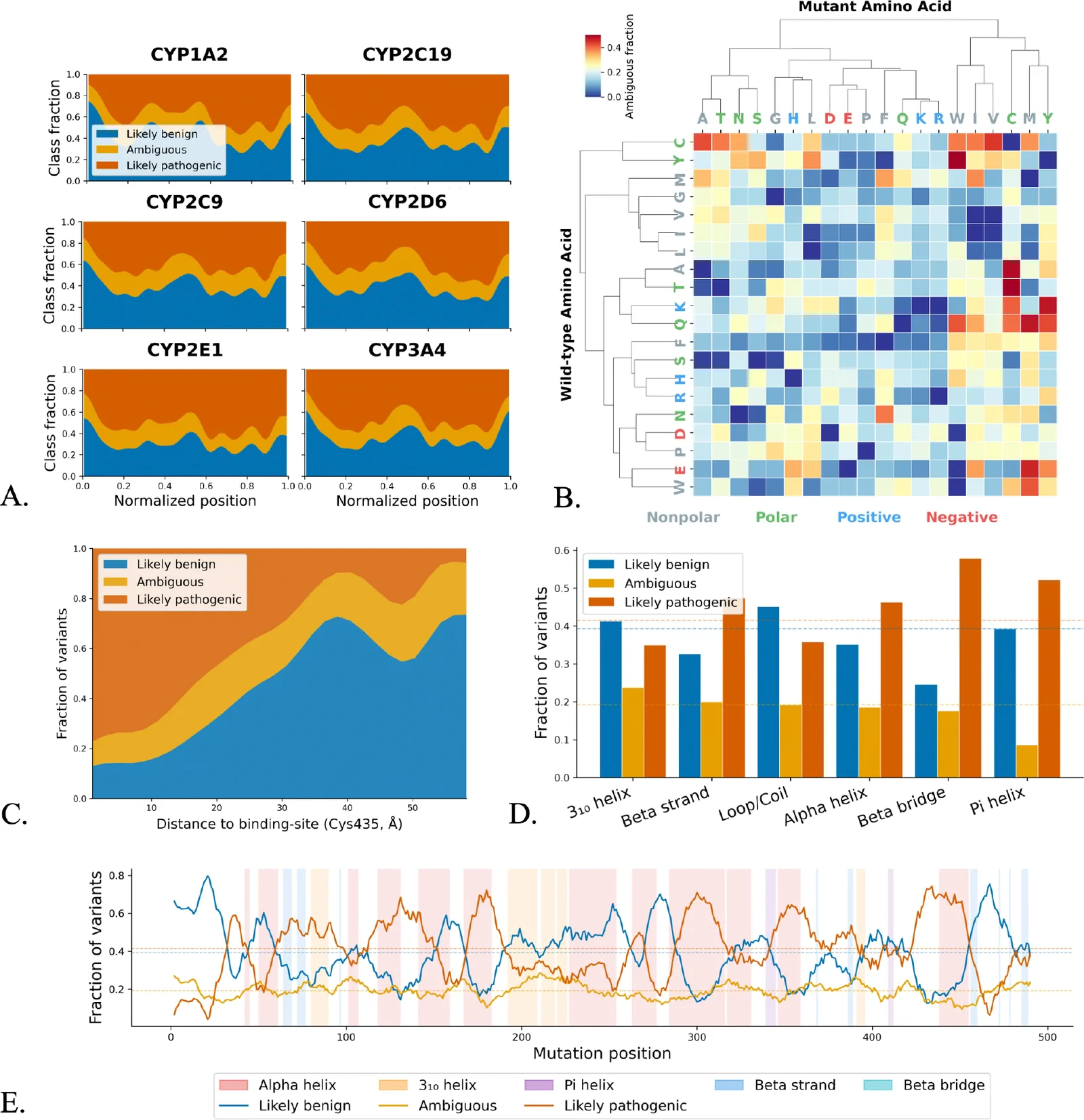
Candidate explanatory features across AM classes (likely benign, ambiguous, likely pathogenic) for all possible missense variants scored by AM. **A.** Class composition versus normalized residue position (0 = N-terminus, 1 = C-terminus; normalized to allow for comparison between CYPs) for the six CYPs (CYP1A2, CYP2C19, CYP2C9, CYP2D6, CYP2E1, CYP3A4). **B.** Ambiguous fraction (ambiguous calls / total) per wild-type → mutant amino acid substitution, pooled across CYPs; rows and columns ordered by hierarchical clustering (Ward linkage), labels colored by biochemical group (nonpolar, grey; polar, green; positive, blue; negative, red). **C.** Class fractions versus C*α* distance (Å) to the binding site (Cys435) in CYP2C9. **D.** Class distribution by secondary-structure type in CYP2C9; dashed lines mark protein-wide per-class averages. **E.** Class fractions along the CYP2C9 sequence (15-residue rolling average), with secondary-structure elements shaded by type.

Positionally, all three AM classes deviate strongly from a uniform distribution along the protein (benign *χ*^2^ = 3334.023,df = 59*,p* < 10^−16^; pathogenic *χ*^2^ = 3659.941*, p* < 10^−16^; ambiguous *χ*^2^ = 419.030*, p* = 1.031 × 10^−55^; Pearson’s *χ*^2^ goodness-of-fit test with BH correction), but the effect is far weaker for the ambiguous class (*χ*^2^*/*df = 7.102) than for the benign and pathogenic classes (*χ*^2^*/*df = 56.509 and 62.033). Thus, benign and pathogenic variants strongly cluster in specific regions, whereas ambiguous variants are comparatively scattered (Fig. 3A).

Substitution chemistry is significantly associated with AM class (*χ*^2^ = 658.319*, p* = 1.117 × 10^−143^, Pearson’s *χ*^2^ test of independence; Fig. 3B), yet the ambiguous fraction differs only marginally between radical and conservative substitutions (18.713% vs. 16.874%; *χ*^2^ = 27.106, *p* = 1.926 × 10^−7^, *ϕ* = 0.022; collapsed 2 × 2 test). The significance therefore reflects statistical power rather than a substantively large effect.

The clearest structural signal comes from distance to the binding site, yet it too bypasses the ambiguous class (Fig. 3C). Per-residue class fraction correlates significantly with *Cα* distance to the heme-coordinating residue Cys435 for likely benign (*ρ* = 0.476*, p* = 4.72 × 10^−29^, two-sided Spearman’s rank correlation) and likely pathogenic (*ρ* = −0.466*, p* = 1.13 × 10^−27^) variants. This means that pathogenic variants enrich near the binding site and benign variants far from it, consistent with proximity to the critical residue driving loss of function. The ambiguous class, however, shows no such association (*ρ* = 0.010*, p* = 0.823).

Secondary structure is likewise associated with AM class (*χ*^2^ = 169.855*, p* = 2.973×10^−31^, *χ*^2^ test of independence; Fig. 3D-E), but the ambiguous enrichment is concentrated in the rarest structural elements. Ambiguous fractions range from 8.612% (*π*-helix, n=209) to 23.736% (3_10_ helix, n=969) against a CYP2C9 baseline of 19.180%, while the dominant categories (*α*-helix, n=3,724; loop/coil, n=3,572) stay within ±0.6% of baseline.

### 3.4. A CYP-specific ensemble recovers most of the missing activity signal

Although AM and ESM-2 are strongly correlated overall (*ρ* = −0.797; Fig. S3A), their agreement is far weaker within the ambiguous class (*ρ* = −0.223) than within the benign (*ρ* = −0.488) or pathogenic (*ρ* = −0.615) classes. The two predictors thus diverge precisely where AM is least informative. Because AM and ESM-2 carry partly independent signal, most notably in the ambiguous class, we reasoned that combining them could recover activity signal that neither captures alone. An AM + ESM-2 ensemble improves overall *ρ* only modestly (0.699) and barely lifts the ambiguous class (0.340; Table 1). The larger gain comes from a CYP2C9-specific KNN over ESM-2 embeddings, which alone reaches an ambiguous-class *ρ* of 0.707. The full AM + ESM-2 + KNN ensemble is best (overall *ρ* = 0.825, ambiguous *ρ* = 0.715; Table 1), closing most of the gap to the other classes (Fig. S3B–C). The learned weights favor KNN (0.21) over ESM-2 (0.07) and AM (0.05), indicating that the CYP-specific signal the KNN reads from ESM-2 embeddings is more informative here than the proteome-generic AM and ESM-2 scores.

### 3.5. Predicted activity does not align with clinical or pharmacogenetic annotations

**Fig. 4.**
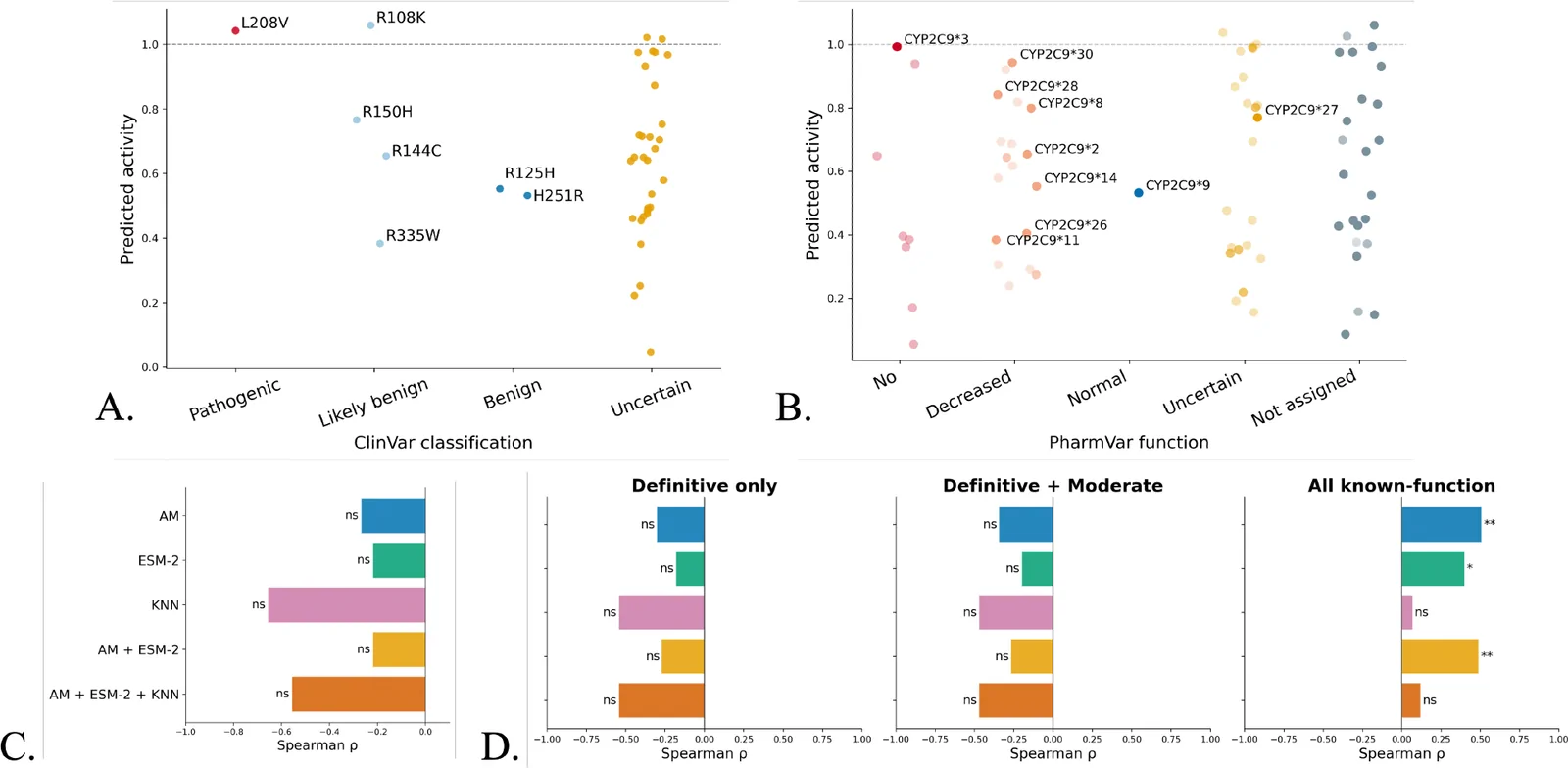
Predicted activity of CYP2C9 missense variants plotted against categorical labels from ClinVar and PharmVar. **A, B.** Predicted CYP2C9 activity (y-axis) versus ClinVar (**A**) or PharmVar (**B**) annotations (x-axis) for the AM + ESM-2 + KNN ensemble model. Each point is a single missense variant; selected variants are labeled. In (**B**), marker opacity encodes PharmVar evidence level: fully opaque markers denote definitive-evidence alleles and progressively more transparent markers denote moderate- and limited-evidence alleles. **C, D.** Spearman correlation of predicted activity with ClinVar (**C**) or PharmVar (**D**) annotation, by model. Significance is indicated per bar, where *p < 0.05; **p < 0.01; ***p < 0.001; ns, not significant. In (**D**), separate correlations are shown at the different PharmVar evidence thresholds (definitive only [n=9], definitive + moderate [n=11], all known function [n=27]).

Recovering biochemical activity does not translate into reliable agreement with CYP2C9 clinical or pharmacogenetic function labels from ClinVar and PharmVar, respectively (Fig. 4A–B). Both label sets are ordinally encoded from no to normal function, so agreement would appear as positive correlation; instead correlations are largely non-significant and, for the best performing models, nominally negative. Against ordinal ClinVar categories the AM + ESM-2 + KNN ensemble model is negatively correlated (*ρ* = −0.558, non-significant), opposite the expected direction. PharmVar shows the same pattern at its highest-confidence tiers (AM + ESM-2 + KNN *ρ* = −0.548 at definitive evidence, non-significant); positive and significant correlations emerge only for AM (*ρ* = 0.509), ESM-2 (*ρ* = 0.399), and AM + ESM-2 (*ρ* = 0.489) once all annotated alleles are considered (Fig. 4C–D), despite the correlation of all KNN-containing models remaining near zero (*ρ* = 0.030–0.121, non-significant). The ensemble that best predicts biochemical activity is thus not the one that best matches clinical labels.

These correlations rest on very few labeled variants, so individual annotations carry large leverage. CYP2C9*3 (I359L) is predicted to retain 79–100% of wild-type activity by every model, diverging not only from its “No Function” PharmVar label (with the highest tier of evidence) but from its measured DMS activity of 45%. L208V, ClinVar’s sole CYP2C9 variant carrying a “pathogenic” clinical-significance assertion, shows a different pattern, with its activity prediction and DMS agreeing (0.89 vs. 0.93) against the clinical label. DMS activity, clinical pathogenicity, and pharmacogenetic function are therefore related but distinct constructs, and accuracy against a biochemical assay does not guarantee concordance with clinical or pharmacogenetic annotations.

### 3.6. Variant effects depend on the substrate

**Fig. 5.**
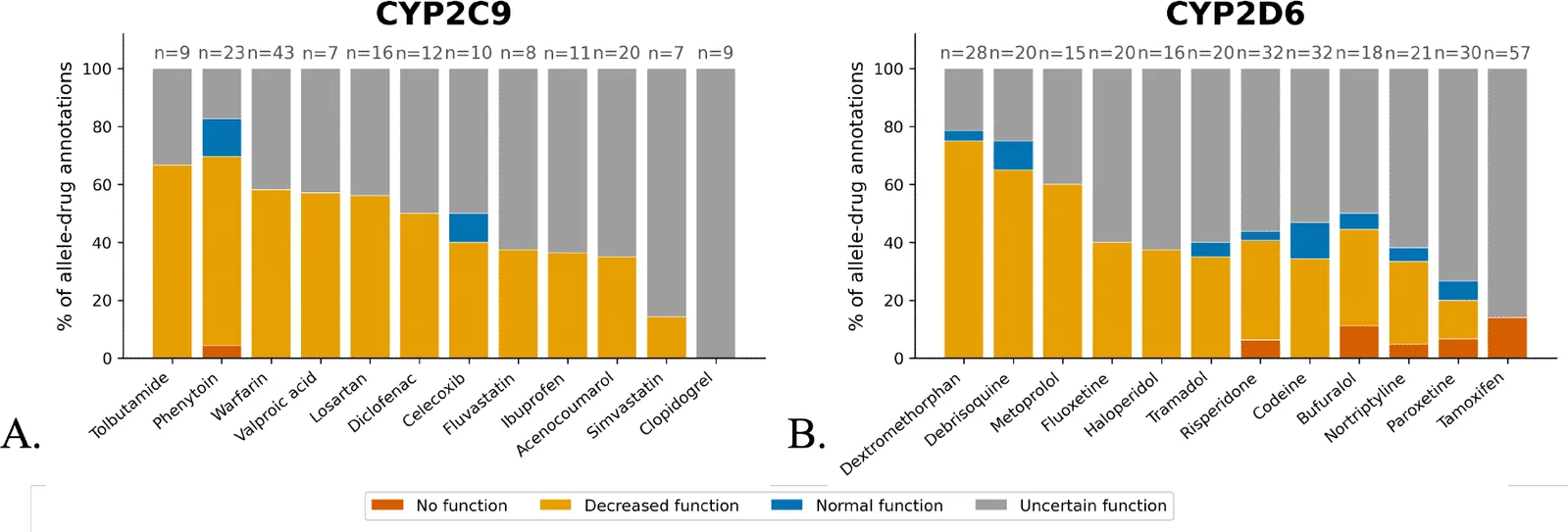
Substrate-resolved variant effects from ClinPGx. Stacked bar charts showing, for two representative CYP genes (CYP2C9 (**A**) and CYP2D6 (**B**)), the distribution of ClinPGx function annotations (no function, decreased function, normal function, and uncertain function) across drug substrates (x-axis). Each bar is one drug and shows the percentage of allele–drug annotations assigned each function label (y-axis, % of allele–drug annotations, 0–100%), where one annotation is a single allele–drug pair. The number of annotations (n) is given above each bar. The equivalent charts for all six CYPs examined are in Fig. S4.

A likely reason no single activity value reconciles these labels from different sources is that, for CYPs, variant effect is not one number. In ClinPGx, the same CYP2C9 variant is annotated with different functional consequences for different drug substrates, and this substrate dependence is seen across the CYPs examined (Fig. 5 for CYP2C9 and CYP2D6; all six CYPs in Fig. S4). Of 185 variants tested against at least two of a gene’s most commonly co-annotated drugs, 24 (13.0%) carried at least two distinct non-uncertain functional labels depending on the substrate, with 14 (58.3%) of these spanning the full range from normal to impaired (no- or decreased-function) activity depending on which drug was tested. Although sparse per-variant annotations left formal testing underpowered, the substantial fraction of substrate-discordant variants shows that substrate identity, held fixed by most current models, is a variable of variant effect that a single activity value cannot capture.

## 4. Discussion

Using CYP pharmacogenes, we found that homology-based prediction fails in a specific, reproducible way: AM and ESM-2 assign no usable activity signal within AM’s intermediate ambiguous class, a failure that sequence position, substitution chemistry, binding-site distance, and secondary structure do not explain. A CYP-specific KNN over ESM-2 embeddings recovers most of this signal, confirming that ESM-2 representations contain relevant information missing in the conservation-derived score. Yet even the highly accurate AM + ESM-2 + KNN ensemble model does not reliably align with clinical or pharmacogenetic labels, and across CYPs the same variant is annotated with different consequences for different drug substrates in ClinPGx, pointing to substrate identity as a feature none of the current predictors capture.

### 4.1. Why homology breaks down on pharmacogenes

Conservation-based predictors succeed when evolutionary tolerance is a good proxy for functional consequence. In CYPs that proxy breaks down: because CYP evolution is tied to xenobiotic breadth rather than a single conserved catalytic output,^3,4,6^ evolutionary tolerance at a position reflects accommodated diversity more than activity toward any particular substrate. The AM ambiguous class is thus not incidental but the score band where the evolutionary signal is genuinely mixed, and our finding that scores there are uninformative about measured activity is what one would expect if the underlying quantity the model encodes has decoupled from catalytic function. The failure of structural and biochemical priors to explain this class is consistent with the same interpretation.

### 4.2. Function is multi-dimensional, and existing ground truth reflects this

A predictor is only as well-posed as its target, and our results make explicit that the resources used to define variant effect are not interchangeable: biochemical activity toward a probe substrate (DMS), disease causation (ClinVar), pharmacogenetic function (PharmVar), and substrate-resolved consequence (ClinPGx) answer different questions. That a model can reproduce measured activity closely yet not align with clinical labels is therefore expected rather than anomalous once function is recognized as multi-dimensional. The recent literature independently supports this. A DMS of CYP2C19 uncovered a substrate-specificity–abundance tradeoff within a single assay,^25^ showing that even one experiment’s notion of function is substrate-contingent; the difficulty of assigning substrate-dependent allelic effects is a standing clinical challenge;^32^ and CYP2C9 activity depends even on cofactor context.^33^ The ceiling that VEPs encounter is therefore as much definitional as methodological, since the target itself is underspecified when a CYP variant’s effect is collapsed to a single substrate-agnostic number.

### 4.3. Relationship to prior computational approaches

Our protein-specific recovery of activity signal fits an active trend of adapting general predictors to narrower problems, from fine-tuning protein language models on DMS data^34–36^ to pharmacogene-specific models that outperform off-the-shelf tools.^20–24^ We extend this work by localizing exactly where proteome-wide models fail (the ambiguous class) rather than reporting aggregate gains, and by showing that even a strong protein-specific model still cannot accurately predict clinical and pharmacogenetic labels — pointing to a ceiling that adaptation alone cannot cross.

While some models do incorporate substrate identity, they remain narrow proofs of principle. Site-of-metabolism and enzyme-kinetics models predict how the wild-type enzyme handles a given drug,^37–42^ but do not predict how a genetic variant alters metabolism of a specific substrate. Conversely, the widely accepted VEPs are substrate-agnostic. Work that does combine the two remains protein- or task-specific.^43,44^ To our knowledge, no widely used, generalizable VEP conditions on the substrate.

### 4.4. Toward substrate-conditioned variant-effect prediction

We therefore propose substrate identity as the leading candidate for the non-homologous variable that current predictors omit, and substrate-conditioned prediction as the path beyond homology. Rather than mapping a variant to a scalar, such a model would predict function for a (variant, substrate) pair, most naturally as substrate-specific kinetic parameters of the kind that enzyme-kinetics models already target for wild-type enzymes.^41,42^ Direct biochemistry confirms this hypothesis: a single active-site substitution can shift CYP2C9 metabolism in opposite directions across substrates. F476W raises (S)-warfarin catalytic efficiency five-fold while lowering (S)-flurbiprofen twenty-fold,^45^ and active-site mutations reorder turnover of warfarin, flurbiprofen, and phenytoin differently.^46^ These corroborate the substrate discordance we observe statistically in ClinPGx: for a multi-substrate enzyme, a variant’s effect is a function of the substrate, which is exactly what a substrate-conditioned predictor must learn. With the biological rationale thus established, the principal remaining obstacle is data: substrate-resolved measurements are sparse, so we present this as a direction rather than a result, though multi-substrate proof-of-concept studies suggest it is increasingly tractable.^47^

The clinical motivation also aligns with this framing. Genotype-guided prescribing is inherently drug-specific,^9,10^ so a substrate-conditioned predictor targets exactly what the clinic requires, whereas a single activity score does not. The most promising route beyond homology here thus runs less through better similarity search than through enriching the definition of function so that prediction is conditioned on substrate in addition to sequence conservation.

### 4.5. Limitations

Several limitations bound these conclusions. The accuracy we recover comes from a protein-specific, recalibrated model whose weights favor the nearest-neighbors component over the proteome-wide scores; it measures how much activity signal homology retains once recalibrated, and is not a general-purpose homology-independent predictor. Our activity benchmark rests on a single DMS screen for one gene.^8^ Combining biochemical, clinical, and pharmacogenetic data is confounded by differences in measurement, thresholds, expression system, and evidence level, leaving generalization to pooled corpora and other CYPs open. The clinical and pharmacogenetic comparisons rest on a small number of labeled variants, so their correlations are non-significant, sensitive to individual labels, and unstable in sign across evidence tiers; we draw no strong statistical conclusions from them. Finally, sparse substrate-resolved annotations in ClinPGx establish that substrate-dependent effects occur for a meaningful fraction of variants but leave their overall prevalence underpowered to estimate.

## Supplementary Material

* Code and supplementary materials are available at https://github.com/hhelenxu/cyp-variant-effect-prediction.

## Figures and Tables

**Table 1.** Predictive performance of individual models and ensembles against CYP2C9 DMS activity. Spearman’s *ρ* between predicted and measured activity, reported overall and within each AM class (benign, ambiguous, pathogenic), for AM, ESM-2, KNN, and the AM + ESM-2 and AM + ESM-2 + KNN ensembles (out-of-fold, 5-fold cross-validation).

Model	Overall *ρ*	Benign *ρ*	Ambiguous *ρ*	Pathogenic *ρ*

AM	0.638	0.339	0.069	0.414
ESM-2	0.678	0.544	0.340	0.445
KNN	0.801	0.629	0.707	0.727
AM + ESM-2	0.699	0.551	0.340	0.481
AM + ESM-2 + KNN	**0.825**	**0.673**	**0.715**	**0.741**
